# Data Publications Correlate with Citation Impact

**DOI:** 10.3389/fnins.2016.00419

**Published:** 2016-09-13

**Authors:** Florian Leitner, Concha Bielza, Sean L. Hill, Pedro Larrañaga

**Affiliations:** ^1^Computational Intelligence Group, Department for Artificial Intelligence, Universidad Politécnica de MadridMadrid, Spain; ^2^Data Catalytics S.L.Madrid, Spain; ^3^Blue Brain Project, Campus BiotechGeneva, Switzerland

**Keywords:** data article citation index, DAC-index, citations, data sharing, open data, data publications

## Abstract

Neuroscience and molecular biology have been generating large datasets over the past years that are reshaping how research is being conducted. In their wake, open data sharing has been singled out as a major challenge for the future of research. We conducted a comparative study of citations of data publications in both fields, showing that the average publication tagged with a data-related term by the NCBI MeSH (Medical Subject Headings) curators achieves a significantly larger citation impact than the average in either field. We introduce a new metric, the data article citation index (**DAC-index**), to identify the most prolific authors among those data-related publications. The study is fully reproducible from an executable Rmd (R Markdown) script together with all the citation datasets. We hope these results can encourage authors to more openly publish their data.

## Introduction

Neuroscience and molecular biology are becoming more data-centric, with increasing numbers of bio-molecule databases, brain atlases, connectomics, and pathway maps, behavioral data, and imaging datasets being published. Due to data's central role in today's high-impact research, the idea that data should be available and shared is—finally (Nelson, 2009)—becoming more popular among policy makers and scientists alike (Sejnowski et al., 2014). The important role of small-scale data in science and several initiatives that collect and provide neuroscience data were described (Ferguson et al., 2014). Further indexing in meta-repositories, such as the NIF (Gardner et al., 2008), NeuroMorpho (Nanda et al., 2015), and NITRC (Luo et al., 2009), and the rise of a new class of publications, such as Scientific Data's data descriptors (Editorial, *Scientific Data* 1, 2014), should all help fellow researchers to stay up-to-date with the current exponential growth of neuroscience data.

Open data (Boulton et al., 2011) leads to better research models because it enables integration of more diverse information, population effects become more robust with larger datasets, and most of all, open data ensures transparency and scientific reproducibility (Walport and Brest, 2011; Milham, 2012; Poldrack and Gorgolewski, 2014). However, sharing data “will require a cultural shift to standardize, integrate, and synthesize diverse types of data” (Sejnowski et al., 2014) and sharing in general has yet to become mainstream (Nelson, 2009). We should be following encouraging examples, such as the positive effect that making the data from the Human Genome Project publicly available had (Yozwiak et al., 2015). Beyond self-control, open data should be made a requirement to receive funding (Bobrow, 2015). New big data initiatives in neuroscience, such as BigNeuron, a community effort to benchmark single-neuron reconstruction algorithms (Peng et al., 2015), would benefit from incentivized sharing, make (more) material available for comparing, evaluating, and developing scalable data processing techniques.

We conducted a statistical study to show that this transformation can be attractive, demonstrating that articles tagged with a MeSH (Medical Subject Heading) data term have a strong correlation with a quantifiable, positive effect on an article's citation impact. Prior to our work, Heather Piwowar, a strong advocate of data sharing, has shown in Piwowar and Vision (2013) and Piwowar et al. (2007) that if gene expression studies publish their raw microarray data, they have a significantly higher citation impact than studies that do not. By leaning on that work, while measuring impact across entire subject areas, we will show that the beneficial effect of publishing data-related articles in fact is detectable across neuroscience and molecular biology as a whole. In our study, we find similar relative increases in citation counts to what Piwowar reported in 2013 for microarray datasets.

## Methods

All citation data were collected from Thomson Reuters' Web of Knowledge (WoK) website, limited to the years 1950–2013, both inclusive. **Neuroscience** citations are restricted to the subject area “SU = (Neurosciences & Neurology).” **Molecular biology** citations are selected as follows: “SU = [(Biochemistry & Molecular Biology OR Life Sciences & Biomedicine) NOT Neurosciences & Neurology].”

**Data-related article** citations were selected by querying for all citations with any database- or atlas-specific Medical Subject Heading (MeSH term), and then intersected with the above subject area queries; That is, we selected all MeSH terms with “Atlases” or “Databases” as major topic—except for “Databases, Bibliographic”—as the *exhaustive* set of MeSH terms that can be used to describe articles that either are collections of facts and data or are publications of public repositories and databases. Therefore, we collectively refer to all articles tagged with one or more of those MeSH-terms as data-*related* articles. The population samples (**random articles**) were selected by randomly choosing a number of PubMed IDs equal to the number of data-related articles *per year*. That is, the random article sets are of the same size and background distribution of years as the data-related articles.

The **author articles** (per-author collections) were selected by downloading all PubMed-specific citations by each author in her respective field (neuroscience or molecular biology). Authors were selected by both their full *and* abbreviated first [and middle] names. That heuristic implies an apparent limitation that names are ambiguous—even full names. For very prolific authors it still provides a highly enriched set as long as there is no collision with another prolific author. For all datasets, the PubMed ID, author names, publication year, and the number of citations per article were downloaded from the WoK website. All these data can be found as 24 tabulated files (^*^.tsv) in the Supplementary Data and their statistical descriptors are found in the Supplementary Material, *Neuroscience citations*, and *Molecular Biology citations* sections.

The ***p*-values** to evaluate this comparative study of citation counts were generated using the nonparametric, distribution-free rank-sum test (also known as the Mann-Whitney or Wilcoxon test), as implemented by the R standard library function *wilcox.test*.

The **data article citation index** (DAC-index) is defined as the sum of logs of the citation counts of all articles published by an author (aggregated by their normalized names):

(1) $$\begin{array}{l} D=\sum \;log\left(n_{i}\right) \end{array}$$

Where *n* is the citation count for the *i*-th data article published by a given author. We abbreviated their first and middle names to normalize the author names (see Supplementary Material, *A data article citation index* for a detailed discussion).

The entire statistical analysis can be studied in detail and fully reproduced from the provided R Markdown script (journal.Rmd) and the result of running that script (e.g., in R Studio) regenerates the Supplementary Material PDF file (after installing the packages “dplyr” and “readr”). Additional details for all methods described can be found in that Supplementary Material file.

## Results

**Figure 1:**
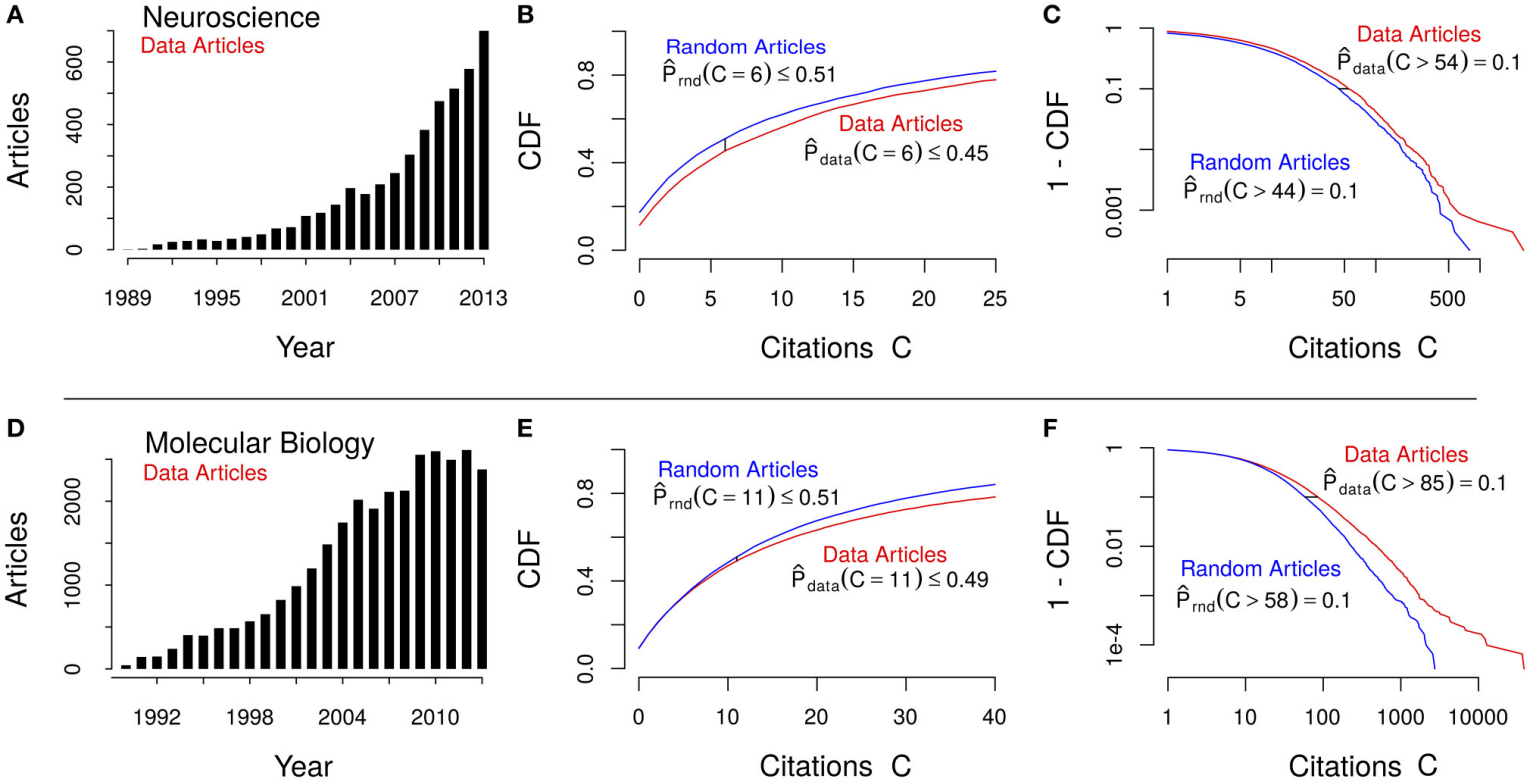
**Comparative study of empirical data *(red)* and random article *(blue)* citation distributions for *neuroscience* (top, A–C) and *molecular biology* (bottom, D–F)**. **Left (A,D)**: Histogram of the number of data articles published per year. **Middle (B,E)**: Cumulative distribution function (CDF) over the first few citation counts C (x-axis), showing the observed probability for the median citation value of average articles (*C* = 6 and *C* = 11, respectively). **Right (C,F)**: Log-log plots of the complementary (1 - CDF) visualizing the growing gap between the data and random citation distributions in the heavy tails. The small horizontal black line marks the observed citation count difference for articles in the top decile (*P* = 0.1; i.e., the 10% most cited articles).

In total, we retrieved the citation counts for 4575 neuroscience and 30,612 molecular biology data and random articles each used in this analysis, as well as two times 10 author sets for the top 10 data authors' citations in either field (according to our data article citation index). For all 20 authors we provide at least 10 data and 10 non-data articles, but most authors have substantially more articles. We compared citation counts for articles that published data or repositories (databases, atlases) against the overall population. Furthermore, we applied the same analysis to molecular biology articles and therefore, can show that the observed phenomenon is not domain-specific. The underlying distribution of publication years for both data-related and random articles are shown in Figures 1A,D.

An immediate observation is that 2.57 times as many data articles are being published in molecular biology than in neuroscience after accounting for population size differences. A graphical analysis shows that citations in the data article sets follow a distribution shifted to the right when compared to their entire field—that is, data articles are likely to receive more citations than the average article.

We found that the *median* number of citations for data articles is larger than the corresponding (random) sample median: For neuroscience, the median citation count of a data article is 8, while the population sample has a median citation count of only 6. For molecular biology, the medians are 12 and 11, respectively. Furthermore, we can confirm that those shifts are statistically highly significant (one-sided rank-sum tests, *p* < < 0.001, for both tests; see *Methods* and Supplementary Material, *Citation count comparisons*). From Figures 1B,E, this difference can be quantified: The proportion of data articles receiving a median number of citations equal to the field's average is 13% (0.51/0.45) and 4.1% (0.51/0.49) larger in neuroscience and molecular biology, respectively.

And from Figures 1C,F, we can measure the relative increase in citations for the most cited decile of articles: 25% (54/44) and 47% (85/58), for neuroscience and molecular biology, respectively. This means that not only are data articles more likely to be cited than an average article, but highly cited data articles receive even more citations than the general population of highly cited articles. However, this observation for highly cited articles is more suggestive for molecular biology than for neuroscience (Figures 1F vs. C).

The top 10 data-publishing authors according to the DAC-index are shown in Table 1. Sixteen out of twenty (80%) of the authors received a statistically significant (*p* < 0.05, one-sided rank-sum test) larger number of citations for their data articles if compared to all their other articles in the same field.

**Table 1:** **DAC-index ranking for the top 10 neuroscience (left) and molecular biology (right) authors**.

***Neuroscience***				**Rank**	***Molecular Biology***			
**Author name**	**DAC-idx**	**Citations**	**Median**		**Author name**	**DAC-idx**	**Citations**	**Median**
*Arthur W. Toga*	113	1192	21 (29)	1	Amos Bairoch	685	22,504	88.5 (52)
Anthony Marmarou	108	2246	80 (26)	2	Eugene V. Koonin	366	21,984	89.5 (55)
Gordon D. Murray	76	1061	81 (33)	3	A. Keith Dunker	308	6187	54.5 (30.5)
Andrew I. R. Maas	68	850	79 (18.5)	4	Richard M. Durbin	305	23,790	148 (78.5)
Ewout W. Steyerberg	67	848	81 (19.5)	5	*Andrej Sali*	272	5463	42 (35)
Juan Lu	66	760	79 (14)	6	Jeffrey Skolnick	254	3743	42 (28)
Michal J. *DeVivo*	62	934	56 (24)	7	Denis F. Hochstrasser	235	3561	55 (22.5)
Isabella Butcher	60	741	79 (18.5)	8	Jean-Charles Sanchez	229	3430	59 (24)
Gillian S. McHugh	60	741	79 (25)	9	*Ron D. Appel*	227	4549	33.5 (32)
*David C. Van Essen*	58	1394	67 (65)	10	Vladimir N. Uversky	223	4984	47 (26.5)

*Data articles except for authors with italic names received significantly more citations than their non-data articles. DAC-idx: DAC-index according to Formula (1). Citations: Authors' cumulative citations on data articles. Median: Author's median number of citations for data articles (in parenthesis: median for all articles by that author)*.

## Discussion

Selecting data-related articles via MeSH terms has important advantages over any selection technique the authors could implement. In other words, it makes identifying data-related publications robust, objective/unbiased, unambiguous, and, most importantly, reproducible (e.g., to easily repeat the study again in a few years and observe changes). A MeSH-based selection strategy ensures an impartial third party selects the data-related articles, which provides a huge credibility advantage over any method designed in-house. MeSH terms are assigned by professional bio-curators that receive special training to make these annotations and are specialists on the set of terms they assign (see the online NLM Medical Subject Headings Fact Sheet). It should further be noted that bio-curation is an area of research all of its own, represented by the Intl. Soc. for Biocuration (biocuration.org) (Gaudet et al., 2012). This MeSH terms-based strategy leads to an admittedly broad selection of articles, but based on the *exhaustive* set of data-related MeSH terms. I.e., not all articles tagged with these headings can be guaranteed to have directly associated datasets, but this approach is an unbiased, impartial approximation of the true set of data articles. And for any fellow researcher, to either reproduce the results or update them at a later stage, it is straightforward to apply the same selection strategy.

It is clearly noticeable that the growth of data publications is more recent in neuroscience (Figures 1A,D) and therefore, had less time to accrue citations; nonetheless, just as many neuroscientists have an increased data citation impact as molecular biologists (8/10 each). A possible explanation for the increased number of data-related articles in molecular biology over neuroscience could be the fact that some journals in molecular biology require authors to submit the related data to public repositories. However, such requirements by molecular biology journals typically only apply to genomic sequences and protein structure data. For example, we know of no *common* requirement for publishing the raw epigenetic and gene expression data of cancer studies and similar phenotype-related investigations.

To increase the amount of data sharing, a promising strategy would be to identify the top data-sharing authors. However, it is not straightforward to deduce data *reuse* from the existing publications and their citation counts: (1) It is not feasible to establish *all* publications that have published data—we only consider the National Library of Medicine-curated subset. (2) We are not aware of a fully deductive approach to author name disambiguation. (3) It is difficult to verify if a citing article actually used the data. Therefore, several initiatives are under way to assign unique identifiers to the datasets themselves that should (only) be cited if the data itself has been reused, e.g., the CODATA-ICSTI Task Group on Data Citation Standards Practices (2013) and the RRID initiative (Bandrowski et al., 2016) or *data papers* for neuroimaging (Gorgolewski et al., 2013) or biodiversity (Chavan and Penev, 2011). In the mean time, by acknowledging the described limitations of our approach, we approximate the true data-sharing index with the data article citation index.

Due to the low volume of data publications (relative to the entire bibliome), using the *de facto* index standard, the h-index, is impractical because authors of highly cited resources that only published very few data articles will be placed among the lowest ranks (Lehmann et al., 2006). On the other hand, if a sum-based index were applied to the citation counts, all (co-) authors of the most cited one or two papers would dominate the top ranks. Therefore, we propose an index based on the sum of citation count logs for MeSH-tagged data papers, the data article citation index (**DAC-index**, see *Methods, formula (1)*. A sum-of-logs based approach to rank authors (in computational linguistics) has also been published by Radev et al. (2015). There, the sum of citation count logs it is used as an auxiliary approach to fix the bias of highly cited authors in citation networks (see Table 11 in Radev et al., 2015).

Only Arthur W. Toga's median data article citation count is lower than his non-data article median (compare Data vs. All in Table 1). Note that this only implies that Toga's “non-data” publications have even more impact than his already impressive data articles—after all, he leads the DAC-index ranking—and does not invalidate our conclusions. In other words, both leaders—Toga and Bairoch—are exceptional cases, even within these top ranks [Amos Bairoch (UniProt) is the only author in the top ten that has (far) more data articles than other, non-data articles]. In neuroscience, Arthur W. Toga represents the LONI-BIRN (Helmer et al., 2011) database, closely followed by Anthony Marmarou [TBI-IMPACT, (Marmarou et al., 2007)] and his co-authors (next 7 ranks), and finally, David Van Essen [NIH-HCP (Van Essen et al., 2012)].

To understand the results, it is important to keep the main limitation of this work in mind: Data-related articles might differ from the bulk of articles in many ways, some of which will independently affect citation count, such as journal or publication type, funding, geographic location, etc. However, by ensuring equal sample sizes over years, the very influential factor of time on citation count was excluded as a confounding variable. Therefore, the study only claims a correlation between the two article populations; it cannot prove a causational effect of publishing data on citations.

## Conclusions

Overall, this study presents *strong evidence* that data-related publications tagged with the described MeSH terms receive a significantly increased number of citations, both in neuroscience and molecular biology as a whole, when compared to their fields' averages. The presented data article citation index (*Methods* and Table 1) could be used to incentivize the publication of data and the use of data citations. Finally, a similar and very strong correlation between citation count and article type can be observed across the collected works of most of the top data-publishing authors.

## Future directions

For journals, one avenue to encourage open data would be requiring authors to publish the raw data together with the article itself: For example, the Journal of Biological Chemistry (JBC) explicitly requires the prior submission of any functional genomic or proteomic data to a public repository (see is.gd/ZKDoRS). Such requirements should be expanded to all data related to a publication, at least where feasible in terms of volume.

For authors, in addition to using open access publication models (Gargouri et al., 2010), we hope this work has further encouraged them to open their data to fellow researchers. But it must be acknowledged that not all scientists have the necessary resources to finance or maintain the required storage facilities needed to provide their data, while openness affects precisely those scientists the most (Evans and Reimer, 2009) (ironically, a closed-access publication). “Free” public data hosting solutions supported by public funds and maintained by the academic world can equalize that limitation, also highlighting the continued importance of supporting scientific databases. Finally, efforts to make data citable (data citations, data papers, data descriptors, RRIDs, etc.) should be expected to make the impact of open data more obvious in the future, especially to measure data reuse.

## Author contributions

FL collected the data, carried out the statistical analyses and wrote the article. CB and PL ensured the statistical validity of the content. CB, SH, and PL corrected and contributed to the article content. CB and PL directed the work.

## Funding

This work was supported by the Spanish Ministry of Economy and Competitiveness through the Cajal Blue Brain (C080020-09; the Spanish partner of the Blue Brain initiative from EPFL) and TIN2013-41592-P projects, by the Regional Government of Madrid through the S2013/ICE-2845-CASI-CAM-CM project, and by the European Union's Horizon 2020 research and innovation programme under grant agreement No. 720270 (Human Brain Project).

### Conflict of interest statement

The authors declare that the research was conducted in the absence of any commercial or financial relationships that could be construed as a potential conflict of interest.
