# Supplementary material for: Data Publications Correlate with Citation Impact
Source: Front Neurosci. 2016 Sep 13;10:419. doi: 10.3389/fnins.2016.00419 (PMC5020078; doi:10.3389/fnins.2016.00419)
Supplement: Supplementary file 1 [file DataSheet1.PDF]

A reproducible, comparative study of data article citations in Neuroscience and Molecular Biology (Supplementary material for "Data publications correlate with citation impact")

Florian Leitner, Concha Bielza, Sean Hill, and Pedro Larrañaga

Summer 2015

Contents

Introduction **1**

Study overview . . . 1

Settings and data . . . 2

Field-specific citation distributions **14**

Article citation distributions . . . 14

Citation count comparisons . . . 14

Author-specific citation distributions **19**

A data article citation index . . . 19

Data author comparisons . . . 24

An online DAC-index **28**

References **30**

Introduction

Study overview

This analysis compares various citation count samples of PubMed citations available from the Thomson Reuters ISI Web of Knowledge (WoK). The analysis is split in two main categories (fields): articles from the field of **Neuroscience** (**NS**) and articles from **Molecular Biology** (**MB**; all articles combined with Life Sciences, but excluding the overlap with Neuroscience articles). Note that there still could be remaining articles for both fields in the general Thomson Reuters database and that are not part of PubMed. However, in the general in the biomedical field, only publications indexed by PubMed are universally acknowledged by all scientific institutions.

For both fields, citation counts for the whole set of *data articles* and a *random sample* with an equal distribution of publication years as in the data articles were collected, as well as 20 (ten per field) sets of *author-specific articles*. Details of how these sets were selected will be explained in *Settings and data*. The fundamental statistical issues that are addressed by this work are:

1. Is there a measurable effect on an article's total citation count when it is a data (i.e., atlas or database) publication? Specifically, is there a statistically significant difference between the distribution of data articles and an equally sized random article sample (of non-data articles, but covering the same number of articles per year)?
2. For any differences we find, can those differences be quantified?
3. Furthermore, who are the most prolific data publishing scientists in their respective fields?
4. And does such an author's data article citation counts significantly diverge from her or his non-data article citation counts?

To address the first two questions, we perform the following analyses: A statistical test establishes if data citations arise from the same distribution as a set of random article citations of the same size in each field. In particular, we establish if the data article citation distributions have a significant shift to the right (are "greater than", i.e., using a one-sided test) when compared to their respective random "baselines". The empirical **Cumulative Distribution Functions** (**CDF**s) of the data and random article citation distributions are plotted and manually inspected to establish the magnitude of any differences. The probability difference of reaching the field's median citation count and the citation count difference for the top 10% most cited articles (the top *decile*) between data and random sets are used to quantify those differences. All this will be addressed in the *Field-specific citation distributions* section.

The last section, *Author-specific citation distributions*, addresses the remaining two questions. A ranking of the most prolific data-publishing Neuroscience and Molecular Biology scientists is established by defining a **data article citation index** (**DAC-index**). For reasons explained below, this DAC-index is defined as the sum of logs of an author's data citations. Finally, for the top ten data-publishing authors (according to that DAC-index) we apply a statistical test to evaluate if their data articles received significantly more citations than their other (non-data) articles. Their median data and other article citation counts are calculated to quantify this difference.

Settings and data

R setup

```
library(dplyr, warn.conflicts=F) # var. data manipulation functions, sample_n
library(readr) # data import
set.seed(17)
par(mfrow=c(1,1))
```

Data article selection

The selection of data articles are citations with specific PubMed **Medical Subject Heading** (**MeSH**) terms, either for databases or atlases (see below). (Note that the intuitively also relevant-seeming MeSH term "Data Collection" is used to tag works *about* data collection, not data collections per se.) There are at least four reasons that favor a MeSH-based approach:

1. A MeSH-based selection represents a robust methodological basis for selecting articles that contain or present data sets, as MeSH assignments are expert curated.
2. This selection strategy is based on an objective source as opposed to a necessarily biased list of articles by a meta-repository, e.g. the Neuroscience Information Framework[1].
3. The approach minimizes any ambiguity in reproducing our findings (e.g., if we had used a statistical article classifier instead), as only an additional curation effort by the **National Library of Medicine** (**NLM**) can introduce (explainable) changes in the datasets.

[1] It might be worthy to mention that for any such alternative strategy, all citation lists were substantially shorter than the MeSH-based list.

4. The strategy can be repeated easily in the future – to be precise, as long as PubMed carries out its PubMed MeSH curation, an endeavor which the NLM has been engaged with for several decades now.

For example, articles tagged with "Databases, Factual" are articles that must refer to "*extensive collections, reputedly complete, of facts and data garnered from material of a specialized subject area and made available for analysis and application.*" We explored the use of a number of other MeSH terms as well, e.g., Magnetic Resonance Imaging, but selecting articles with any other term produces substantial numbers of articles that do not publish data, as their definitions do not require the presence of data and articles might just discuss MRI techniques. However, while we could not identify further MeSH terms that could serve as indicators of data articles, this cannot be a claim that our list is exhaustive. Note that we have purposefully excluded "Databases, Bibliographic" from the Databases terms, as that MeSH term covers works that are not necessarily data-related, but rather reference other scientifc works.

Article queries were ran against Thomson Reuter's ISI WoK, limiting the retrieval to the years 1950 to 2013 (inclusive), and using the following expressions to produce the stated result sizes:

```
## Data articles selection
1: MH=(Databases, Chemical OR
       Databases, Factual OR
       Databases, Genetic OR
       Databases, Nucleic Acid OR
       Databases, Pharmaceutical OR
       Databases, Protein OR
       Atlases as Topic)
#> 66K data articles

## Neuroscience article selection
2: SU=(Neurosciences & Neurology)
#> 2.545M NS articles

## Molecular Biology article selection
3: SU=((Biochemistry & Molecular Biology OR
        Life Sciences & Biomedicine) NOT
      Neurosciences & Neurology)
#> 6.493M MB articles

## Neuroscience data article selection
4: #1 AND #2
#> 4,575 NS data articles

## Molecular Biology data article selection
5: #1 AND #3
#> 30,612 MB data articles
```

The **random articles** are selected by limiting the query to randomly selected PubMed IDs proportional to the number of data article citations for each year and category (NS, MB). For example, if there are 200 NS *data* articles for some year, 1000 random PubMed IDs for the same year are randomly sampled from our local PubMed database and the corresponding *NS* citations downloaded. This convoluted procedure is required because our local PubMed mirror (from the NLM) does not contain the subject field assignments of the Thomson Reuter's WoK (to MB and/or NS). In other words, we first select far more random PubMed IDs than necessary to cover a given year but then download only those articles that are assigned to the relevant field.

For the nature of this study, it is noteworthy that the random sets can include (randomly selected) data articles by chance. This is required to allow us to quantify the average citation impact of data articles relative

to the average citation impact of the entire field. Therefore, the random samples are made over the whole set of articles in the field and may contain data articles as well. (And indeed, if the respective two sets we provide are compared, tiny overlaps between the data and random article sets can be found.)

As will be shown, from the set of data articles in each field we then establish a ranking of the most prolific data-publishing authors by defining a DAC-index. Therefore, **author articles** for each of the top ten data-publishing authors (established by that DAC-index) are selected by querying for the respective author name. These queries rely on the WoK advanced query field `AU` and use both an author's abbreviated and full name. All articles that intersect with the relevant field-specific set for that author (i.e., either NS or MB) are downloaded.

Neuroscience citations

The resulting data and a random article set and the ten author-specific article sets have the following statistical descriptors.

- TC - times cited (citation count)
- PY - publication year
- PM - PubMed ID[2]
- AU - author names

```
NS.data <- read_tsv('neurosci.data.tsv')
```

```
## Parsed with column specification:
## cols(
##   TC = col_integer(),
##   PY = col_integer(),
##   PM = col_integer(),
##   AU = col_character()
## )
```

```
summary(NS.data)
```

```
##        TC                 PY             PM                  AU
##  Min.   :   0.00   Min.   :1964   Min.   :  357457   Length:4575
##  1st Qu.:   2.00   1st Qu.:2005   1st Qu.:16328768   Class :character
##  Median :   8.00   Median :2009   Median :19834022   Mode  :character
##  Mean   :  23.11   Mean   :2008   Mean   :18648030
##  3rd Qu.:  22.00   3rd Qu.:2012   3rd Qu.:22243704
##  Max.   :2619.00   Max.   :2013   Max.   :24600800
```

```
NS.rnd <- read_tsv('neurosci.random.tsv')
```

```
## Parsed with column specification:
## cols(
##   TC = col_integer(),
##   PY = col_integer(),
##   PM = col_integer(),
##   AU = col_character()
## )
```

[2] N.B. despite summarized here as a discrete variable here, that has no impact on the study.

```r
summary(NS.rnd)
```

```
##        TC                 PY             PM                  AU
##  Min.   :   0.00   Min.   :1964   Min.   :  125556   Length:7304
##  1st Qu.:   1.00   1st Qu.:2006   1st Qu.:16924483   Class :character
##  Median :   6.00   Median :2009   Median :19782472   Mode  :character
##  Mean   :  16.93   Mean   :2008   Mean   :18749723
##  3rd Qu.:  17.00   3rd Qu.:2012   3rd Qu.:22235782
##  Max.   :1023.00   Max.   :2013   Max.   :24941716
```

```r
NS.aut <- list(Butcher=read_tsv('neurosci.butcher.tsv'))
```

```
## Parsed with column specification:
## cols(
##   TC = col_integer(),
##   PY = col_integer(),
##   PM = col_integer(),
##   AU = col_character()
## )
```

```r
summary(NS.aut[["Butcher"]])
```

```
##        TC               PY             PM                  AU
##  Min.   :  3.00   Min.   :1986   Min.   : 2951184   Length:26
##  1st Qu.: 12.75   1st Qu.:2007   1st Qu.:17375992   Class :character
##  Median : 34.50   Median :2008   Median :18631321   Mode  :character
##  Mean   : 55.15   Mean   :2008   Mean   :18692641
##  3rd Qu.: 80.00   3rd Qu.:2011   3rd Qu.:21032852
##  Max.   :218.00   Max.   :2013   Max.   :24139680
```

```r
NS.aut[["DeVivo"]] <- read_tsv('neurosci.devivo.tsv')
```

```
## Parsed with column specification:
## cols(
##   TC = col_integer(),
##   PY = col_integer(),
##   PM = col_integer(),
##   AU = col_character()
## )
```

```r
summary(NS.aut[["DeVivo"]])
```

```
##        TC               PY             PM                  AU
##  Min.   :  0.00   Min.   :1979   Min.   :  492764   Length:99
##  1st Qu.: 12.00   1st Qu.:1992   1st Qu.: 7255136   Class :character
##  Median : 26.00   Median :1999   Median :10569446   Mode  :character
##  Mean   : 43.03   Mean   :1998   Mean   :10715413
##  3rd Qu.: 56.00   3rd Qu.:2004   3rd Qu.:15512332
##  Max.   :352.00   Max.   :2012   Max.   :23100450
```

```r
NS.aut[["Lu"]] <- read_tsv('neurosci.lu.tsv')
```

```
## Parsed with column specification:
## cols(
##   TC = col_integer(),
##   PY = col_integer(),
##   PM = col_integer(),
##   AU = col_character()
## )
```

```r
summary(NS.aut[["Lu"]])
```

```
##        TC              PY             PM                 AU
##  Min.   :  0.0   Min.   :1989   Min.   : 1325239   Length:181
##  1st Qu.:  6.0   1st Qu.:1998   1st Qu.: 9657549   Class :character
##  Median : 16.0   Median :2002   Median :12421340   Mode  :character
##  Mean   : 39.5   Mean   :2003   Mean   :13953887
##  3rd Qu.: 44.0   3rd Qu.:2009   3rd Qu.:19118603
##  Max.   :377.0   Max.   :2013   Max.   :24391036
```

```r
NS.aut[["Maas"]] <- read_tsv('neurosci.maas.tsv')
```

```
## Parsed with column specification:
## cols(
##   TC = col_integer(),
##   PY = col_integer(),
##   PM = col_integer(),
##   AU = col_character()
## )
```

```r
summary(NS.aut[["Maas"]])
```

```
##        TC               PY             PM                 AU
##  Min.   :  0.00   Min.   :2002   Min.   :11787492   Length:86
##  1st Qu.:  9.00   1st Qu.:2006   1st Qu.:16441528   Class :character
##  Median : 24.50   Median :2008   Median :18402754   Mode  :character
##  Mean   : 46.72   Mean   :2008   Mean   :18673923
##  3rd Qu.: 63.25   3rd Qu.:2011   3rd Qu.:21274122
##  Max.   :320.00   Max.   :2013   Max.   :24139680
```

```r
NS.aut[["McHugh"]] <- read_tsv('neurosci.mchugh.tsv')
```

```
## Parsed with column specification:
## cols(
##   TC = col_integer(),
##   PY = col_integer(),
##   PM = col_integer(),
##   AU = col_character()
## )
```

```r
summary(NS.aut[["McHugh"]])
```

```
##        TC               PY             PM                  AU
##  Min.   :  4.00   Min.   :2007   Min.   :17375988   Length:21
##  1st Qu.: 25.00   1st Qu.:2007   1st Qu.:17375993   Class :character
##  Median : 44.00   Median :2008   Median :18578634   Mode  :character
##  Mean   : 64.86   Mean   :2009   Mean   :19199758
##  3rd Qu.: 84.00   3rd Qu.:2010   3rd Qu.:20156956
##  Max.   :218.00   Max.   :2013   Max.   :24139680
```

```r
NS.aut[["Marmarou"]] <- read_tsv('neurosci.marmarou.tsv')
```

```
## Parsed with column specification:
## cols(
##   TC = col_integer(),
##   PY = col_integer(),
##   PM = col_integer(),
##   AU = col_character()
## )
```

```r
summary(NS.aut[["Mermarou"]])
```

```
## Length  Class   Mode
##      0   NULL   NULL
```

```r
NS.aut[["Murray"]] <- read_tsv('neurosci.murray.tsv')
```

```
## Parsed with column specification:
## cols(
##   TC = col_integer(),
##   PY = col_integer(),
##   PM = col_integer(),
##   AU = col_character()
## )
```

```r
summary(NS.aut[["Murray"]])
```

```
##        TC                PY             PM                  AU
##  Min.   :   0.00   Min.   :1983   Min.   : 2037083   Length:99
##  1st Qu.:  13.50   1st Qu.:1999   1st Qu.:10623064   Class :character
##  Median :  38.00   Median :2006   Median :16958582   Mode  :character
##  Mean   :  84.48   Mean   :2003   Mean   :15002249
##  3rd Qu.:  85.00   3rd Qu.:2010   3rd Qu.:20298196
##  Max.   :1759.00   Max.   :2013   Max.   :24139680
```

```r
NS.aut[["Steyerb."]] <- read_tsv('neurosci.steyerberg.tsv')
```

```
## Parsed with column specification:
## cols(
```

```
##   TC = col_integer(),
##   PY = col_integer(),
##   PM = col_integer(),
##   AU = col_character()
## )
```

```r
summary(NS.aut[["Steyerb."]])
```

```
##        TC               PY             PM                 AU
##  Min.   :  0.00   Min.   :1994   Min.   : 7489218   Length:101
##  1st Qu.: 10.00   1st Qu.:2004   1st Qu.:15335110   Class :character
##  Median : 23.00   Median :2007   Median :17634755   Mode  :character
##  Mean   : 39.09   Mean   :2007   Mean   :17283688
##  3rd Qu.: 52.00   3rd Qu.:2010   3rd Qu.:20814011
##  Max.   :223.00   Max.   :2013   Max.   :24139680
```

```r
NS.aut[["Toga"]] <- read_tsv('neurosci.toga.tsv')
```

```
## Parsed with column specification:
## cols(
##   TC = col_integer(),
##   PY = col_integer(),
##   PM = col_integer(),
##   AU = col_character()
## )
```

```r
summary(NS.aut[["Toga"]])
```

```
##        TC                PY             PM                 AU
##  Min.   :   0.00   Min.   :1979   Min.   :  469960   Length:553
##  1st Qu.:  10.00   1st Qu.:2004   1st Qu.:15261329   Class :character
##  Median :  29.00   Median :2008   Median :18512163   Mode  :character
##  Mean   :  58.39   Mean   :2007   Mean   :17448806
##  3rd Qu.:  60.00   3rd Qu.:2011   3rd Qu.:21304146
##  Max.   :1637.00   Max.   :2013   Max.   :24683973
```

```r
NS.aut[["Van Essen"]] <- read_tsv('neurosci.van_essen.tsv')
```

```
## Parsed with column specification:
## cols(
##   TC = col_integer(),
##   PY = col_integer(),
##   PM = col_integer(),
##   AU = col_character()
## )
```

```r
summary(NS.aut[["Van Essen"]])
```

```
##        TC                PY             PM                 AU
##  Min.   :   0.00   Min.   :1973   Min.   :  120129   Length:140
```

```
##  1st Qu.:  25.75   1st Qu.:1990   1st Qu.: 6531636   Class :character
##  Median :  65.00   Median :1999   Median :10797508   Mode  :character
##  Mean   : 183.24   Mean   :1998   Mean   :11888799
##  3rd Qu.: 198.75   3rd Qu.:2007   3rd Qu.:18094724
##  Max.   :3281.00   Max.   :2013   Max.   :24683992
```

Molecular Biology citations

Molecular Biology (MB) articles include Life Science, but exclude Neuroscience (NS) articles. The resulting data and a random article set and the ten author-specific article sets have the following statistical descriptors (particularly, publication years and citation counts are of interest).

- TC - times cited (citation count)
- PY - publication year
- PM - PubMed ID[3]
- AU - author names

```
MB.data <- read_tsv('molbio.data.tsv')
```

```
## Parsed with column specification:
## cols(
##   TC = col_integer(),
##   PY = col_integer(),
##   PM = col_integer(),
##   AU = col_character()
## )
```

```
summary(MB.data)
```

```
##        TC                 PY             PM                 AU
##  Min.   :    0.00   Min.   :1975   Min.   : 1194384   Length:30612
##  1st Qu.:    3.00   1st Qu.:2003   1st Qu.:14527528   Class :character
##  Median :   12.00   Median :2007   Median :17885655   Mode  :character
##  Mean   :   44.06   Mean   :2006   Mean   :17116994
##  3rd Qu.:   34.00   3rd Qu.:2010   3rd Qu.:20952426
##  Max.   :38693.00   Max.   :2013   Max.   :25145244
```

```
MB.rnd <- read_tsv('molbio.random.tsv')
```

```
## Parsed with column specification:
## cols(
##   TC = col_integer(),
##   PY = col_integer(),
##   PM = col_integer(),
##   AU = col_character()
## )
```

[3] N.B. despite summarized here as a discrete variable here, that has no impact on the study.

```r
summary(MB.rnd)
```

```
##        TC               PY             PM                 AU
##  Min.   :   0.0   Min.   :1966   Min.   : 1280702   Length:34996
##  1st Qu.:   3.0   1st Qu.:2003   1st Qu.:14578001   Class :character
##  Median :  11.0   Median :2007   Median :17637019   Mode  :character
##  Mean   :  26.2   Mean   :2006   Mean   :17054880
##  3rd Qu.:  27.0   3rd Qu.:2010   3rd Qu.:20853271
##  Max.   :2743.0   Max.   :2013   Max.   :24712276
```

```r
MB.aut <- list(Appel=read_tsv('molbio.appel.tsv'))
```

```
## Parsed with column specification:
## cols(
##   TC = col_integer(),
##   PY = col_integer(),
##   PM = col_integer(),
##   AU = col_character()
## )
```

```r
summary(MB.aut[["Appel"]])
```

```
##        TC                PY             PM                 AU
##  Min.   :   0.00   Min.   :1988   Min.   : 1802690   Length:78
##  1st Qu.:  16.50   1st Qu.:1996   1st Qu.: 8998564   Class :character
##  Median :  33.50   Median :1999   Median :10602264   Mode  :character
##  Mean   :  85.68   Mean   :2000   Mean   :11700010
##  3rd Qu.:  86.75   3rd Qu.:2004   3rd Qu.:15249126
##  Max.   :1373.00   Max.   :2009   Max.   :19391179
```

```r
MB.aut[["Bairoch"]] <- read_tsv('molbio.bairoch.tsv')
```

```
## Parsed with column specification:
## cols(
##   TC = col_integer(),
##   PY = col_integer(),
##   PM = col_integer(),
##   AU = col_character()
## )
```

```r
summary(MB.aut[["Bairoch"]])
```

```
##        TC               PY             PM                 AU
##  Min.   :   0.0   Min.   :1982   Min.   : 1286669   Length:157
##  1st Qu.:  26.0   1st Qu.:1995   1st Qu.: 8506147   Class :character
##  Median :  77.0   Median :1999   Median :10356335   Mode  :character
##  Mean   : 187.9   Mean   :2000   Mean   :11637757
##  3rd Qu.: 226.0   3rd Qu.:2004   3rd Qu.:15608167
##  Max.   :1670.0   Max.   :2013   Max.   :23353650
```

```r
MB.aut[["Dunker"]] <- read_tsv('molbio.dunker.tsv')
```

```
## Parsed with column specification:
## cols(
##   TC = col_integer(),
##   PY = col_integer(),
##   PM = col_integer(),
##   AU = col_character()
## )
```

```r
summary(MB.aut[["Dunker"]])
```

```
##        TC               PY             PM                  AU
##  Min.   :  0.00   Min.   :1969   Min.   :   36395   Length:178
##  1st Qu.: 12.25   1st Qu.:1999   1st Qu.:10681844   Class :character
##  Median : 40.00   Median :2006   Median :16667782   Mode  :character
##  Mean   : 95.33   Mean   :2001   Mean   :14591739
##  3rd Qu.: 94.00   3rd Qu.:2009   3rd Qu.:19592405
##  Max.   :986.00   Max.   :2013   Max.   :23758675
```

```r
MB.aut[["Durbin"]] <- read_tsv('molbio.durbin.tsv')
```

```
## Parsed with column specification:
## cols(
##   TC = col_integer(),
##   PY = col_integer(),
##   PM = col_integer(),
##   AU = col_character()
## )
```

```r
summary(MB.aut[["Durbin"]])
```

```
##        TC                 PY             PM                  AU
##  Min.   :    0.00   Min.   :1960   Min.   : 1302004   Length:108
##  1st Qu.:   29.75   1st Qu.:1998   1st Qu.:10571391   Class :character
##  Median :   98.00   Median :2004   Median :14911374   Mode  :character
##  Mean   :  362.29   Mean   :2002   Mean   :14574111
##  3rd Qu.:  243.75   3rd Qu.:2008   3rd Qu.:18998185
##  Max.   :11214.00   Max.   :2013   Max.   :24104757
```

```r
MB.aut[["Hochstr."]] <- read_tsv('molbio.hochstrasser.tsv')
```

```
## Parsed with column specification:
## cols(
##   TC = col_integer(),
##   PY = col_integer(),
##   PM = col_integer(),
##   AU = col_character()
## )
```

```r
summary(MB.aut[["Hochstr."]])
```

```
##        TC               PY             PM                  AU
##  Min.   :  0.00   Min.   :1988   Min.   : 1281090   Length:192
##  1st Qu.: 11.00   1st Qu.:1996   1st Qu.: 8906833   Class :character
##  Median : 25.00   Median :1999   Median :10610496   Mode  :character
##  Mean   : 58.62   Mean   :2001   Mean   :12290706
##  3rd Qu.: 68.00   3rd Qu.:2006   3rd Qu.:16773459
##  Max.   :459.00   Max.   :2013   Max.   :23954032
```

```r
MB.aut[["Koonin"]] <- read_tsv('molbio.koonin.tsv')
```

```
## Parsed with column specification:
## cols(
##   TC = col_integer(),
##   PY = col_integer(),
##   PM = col_integer(),
##   AU = col_character()
## )
```

```r
summary(MB.aut[["Koonin"]])
```

```
##        TC                PY             PM                  AU
##  Min.   :    0.0   Min.   :1983   Min.   : 1317076   Length:500
##  1st Qu.:   21.0   1st Qu.:1996   1st Qu.: 8796420   Class :character
##  Median :   58.0   Median :2001   Median :11446540   Mode  :character
##  Mean   :  135.7   Mean   :2001   Mean   :12538060
##  3rd Qu.:  129.5   3rd Qu.:2006   3rd Qu.:16902967
##  Max.   :11197.0   Max.   :2013   Max.   :24012761
```

```r
MB.aut[["Sali"]] <- read_tsv('molbio.sali.tsv')
```

```
## Parsed with column specification:
## cols(
##   TC = col_integer(),
##   PY = col_integer(),
##   PM = col_integer(),
##   AU = col_character()
## )
```

```r
summary(MB.aut[["Sali"]])
```

```
##        TC               PY             PM                  AU
##  Min.   :   0.0   Min.   :1970   Min.   :   72956   Length:279
##  1st Qu.:  13.0   1st Qu.:1999   1st Qu.:10601956   Class :character
##  Median :  37.0   Median :2006   Median :16507877   Mode  :character
##  Mean   : 100.7   Mean   :2004   Mean   :15180335
##  3rd Qu.:  90.5   3rd Qu.:2010   3rd Qu.:20506463
##  Max.   :5854.0   Max.   :2013   Max.   :24197012
```

```r
MB.aut[["Sanchez"]] <- read_tsv('molbio.sanchez.tsv')
```

```
## Parsed with column specification:
## cols(
##   TC = col_integer(),
##   PY = col_integer(),
##   PM = col_integer(),
##   AU = col_character()
## )
```

```r
summary(MB.aut[["Sanchez"]])
```

```
##        TC               PY             PM                 AU
##  Min.   :  0.00   Min.   :1992   Min.   : 1281090   Length:174
##  1st Qu.: 13.00   1st Qu.:1997   1st Qu.: 9504809   Class :character
##  Median : 28.00   Median :2001   Median :11452661   Mode  :character
##  Mean   : 65.26   Mean   :2002   Mean   :13235713
##  3rd Qu.: 75.75   3rd Qu.:2007   3rd Qu.:17310662
##  Max.   :503.00   Max.   :2013   Max.   :23954032
```

```r
MB.aut[["Skolnick"]] <- read_tsv('molbio.skolnick.tsv')
```

```
## Parsed with column specification:
## cols(
##   TC = col_integer(),
##   PY = col_integer(),
##   PM = col_integer(),
##   AU = col_character()
## )
```

```r
summary(MB.aut[["Skolnick"]])
```

```
##        TC              PY             PM                 AU
##  Min.   :  0.0   Min.   :1985   Min.   : 1293893   Length:203
##  1st Qu.: 12.5   1st Qu.:1997   1st Qu.: 9468208   Class :character
##  Median : 31.0   Median :2003   Median :12609858   Mode  :character
##  Mean   : 50.1   Mean   :2002   Mean   :13361527
##  3rd Qu.: 57.5   3rd Qu.:2008   3rd Qu.:18169078
##  Max.   :586.0   Max.   :2013   Max.   :24204237
```

```r
MB.aut[["Uversky"]] <- read_tsv('molbio.uversky.tsv')
```

```
## Parsed with column specification:
## cols(
##   TC = col_integer(),
##   PY = col_integer(),
##   PM = col_integer(),
##   AU = col_character()
## )
```

```r
summary(MB.aut[["Uversky"]])
```

```
##        TC               PY            PM                  AU
##  Min.   :  0.00   Min.   :1990   Min.   : 1287658   Length:301
##  1st Qu.: 11.00   1st Qu.:2003   1st Qu.:12614167   Class :character
##  Median : 29.00   Median :2007   Median :17578581   Mode  :character
##  Mean   : 72.36   Mean   :2006   Mean   :16895343
##  3rd Qu.: 73.00   3rd Qu.:2010   3rd Qu.:20889377
##  Max.   :983.00   Max.   :2013   Max.   :24072065
```

Article set size comparisons

Neuroscience has 1 database article per 546 publications. Molecular Biology has 1 such article per 212 publications. Therefore, and after adjusting for the absolute sizes of the existing literature in each field, this indicates that there are more than two-and-a-half (2.57) as many database publications in Molecular Biology (incl. Life Science, excl. Neuroscience) as there are in Neuroscience.

Field-specific citation distributions

Article citation distributions

The earliest model for discrete citation count per article was the log-normal distribution (Shockley 1957). Historically, citation counts have been also fitted to power law distributions[4], such as Zipf's law[5], and, in particular, Pareto's law[6], for example in (Solla Price 1965) or (Redner 1998). However, citation counts only exhibit power-law behavior on the most cited articles that have accumulated unusually large numbers of citations. Therefore, a number of other distributions have been suggested, including stretched- and q-exponential distributions (Wallace, Larivière, and Gingras 2009). The only fits that has been reported *with* statistically *significant* goodness-of-fit tests over the *entire* range of citation counts (i.e., $[0, \infty)$) to our best knowledge, however, are log-normal distributions (Stringer, Sales-Pardo, and Nunes Amaral 2008); There, it has been shown that a citation distribution is log-normal if the article set is restricted to a single year and journal. Nonetheless, as stated by Stringer, Sales-Pardo, and Amaral (2010), joining several independent log-normal distributions can result in a distribution that approaches power-law behavior, at least for the subset of highly cited articles. However, for the issues being addressed here, establishing the exact, underlying distribution is not particularly relevant: By relying on the nonparametric, distribution-free (Mann-Whitney aka. Wilcoxon) rank-sum test, we refrain from providing a conclusive answer to this issue.

Citation count comparisons

The most important potential bias when comparing citation count distributions is the underlying distribution of publication years, because older publications are more likely to have accrued more citations simply due to age. Therefore, the number of random articles per year should be exactly the same number of articles as that of the data articles. Note, however, that we decided to not provide perfect matches for the early years before 1990, as this only affects three articles in MB and 18 in NS and therefore has a negligible effect. In

[4] $y \sim x^{-\alpha}$ where x here would be the citation count and y an article's cumulative probability of achieving at least less than that number of citations (i.e., $y = 1$ at $x = 1$, because any article will have at least zero citations); Therefore, it is a CDF where α is known as the power law slope.

[5] $y \sim r^{-\beta}$ with y being an article's citation count and r the article's rank (i.e., order wrt. an article's number of citations) with any $\beta > 0$ that usually is close to unity.

[6] $y \sim x^{\kappa}$ with y being the proportion of articles with a citation count $\geq x$; This complementary CDF that can be associated to the power law by setting $\alpha = 1 + \kappa$.

addition, for NS we choose random articles from a pool where most are from the same two years when most of those 18 data articles were published (i.e., 1974-5); For MB, we select three random articles over the entire relevant period (1966-89). Histograms of the number of data publications per year in each field, covering the statistically relevant years after 1989, are shown in Figures 1 and 2.

```
SampleYears <- function(data.orig, data.sample, year) {
  # sample the same number of cases from data.sample as are in data.orig per year
  # 1. sample the same number of instances from data.sample
  # as are in data.orig for year "yr"
  sample_y <- function(yr, orig, sample)
    sample_n(sample[sample$PY == yr,], nrow(orig[orig$PY == yr,]))
  # 2. sample the same number of instances from data.sample
  # as there are in data.orig for all years before "year"
  base = sample_n(data.sample[data.sample$PY < year,],
                  nrow(data.orig[data.orig$PY < year,]))
  # 3. combine the rows from base with the rows for each later year
  rbind(base, Reduce(function(...) merge(..., all=T),
                     lapply(year:2013, function (yr)
                       sample_y(yr, data.orig, data.sample))))
}
```

```
PlotYears <- function(years, title="Neuroscience") {
  # plot a histogram of the number of articles/year
  y.table = table(years)
  unique.years = unique(years)
  plot(y.table, type="h", lend=1, lwd=7, xlab="Year",
       xaxt="n", yaxt="n", ylab="N. Articles", frame.plot = F)
  axis(1, at=seq(1989, max(unique.years) + ((max(unique.years) - 1989) %% 3), 3),
       tck=-0.025, cex.axis=0.75, lty=1, lwd=1)
  axis(2, at=seq(0, max(y.table), round(max(y.table) / 500) * 100),
       cex.axis=0.75, tck=-0.025)
  text(x=min(years) + 7, y=max(y.table) - 100, label=title)
}
```

```
NS.rnd = SampleYears(NS.data, NS.rnd, 1990)
#CompareYears(NS.data$PY, NS.rnd$PY)
PlotYears(NS.data$PY[NS.data$PY>1988])
```

```
MB.rnd = SampleYears(MB.data, MB.rnd, 1990)
#CompareYears(MB.data$PY, MB.rnd$PY, title="Molecular Biology")
PlotYears(MB.data$PY[MB.data$PY>1988], title="Molecular Biology")
```

The random sampling procedure shown above produces an equally-sized random article set with an equal background distribution of years as the data article set.

Note that the mean is a poor choice to describe the average of a set of citation counts, because the true mean is not the population mean for a non-normal, highly skewed citation distribution with extreme values - e.g. (Wang, Song, and Barabási 2013). Therefore, instead, we use the *median* to describe the average citation counts of our sets. For NS, we observe medians of 8 and 6 for the data and random article citation sets, respectively. For MB, we observe medians of 12 and 11 for the data and random article citation sets, respectively. In other words, the median citation count of (average) data articles is one to two counts higher than that of average (random) articles in their field. (While we never encountered this phenomena, note that

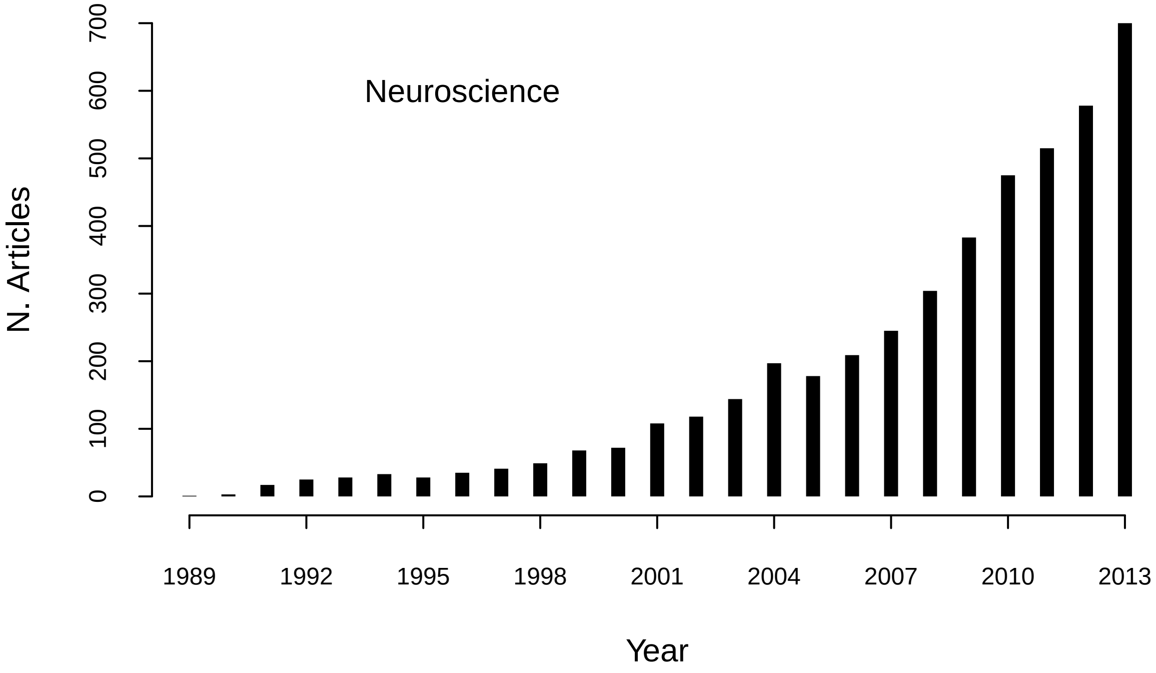

Figure 1: Neuroscience data/random articles per year.

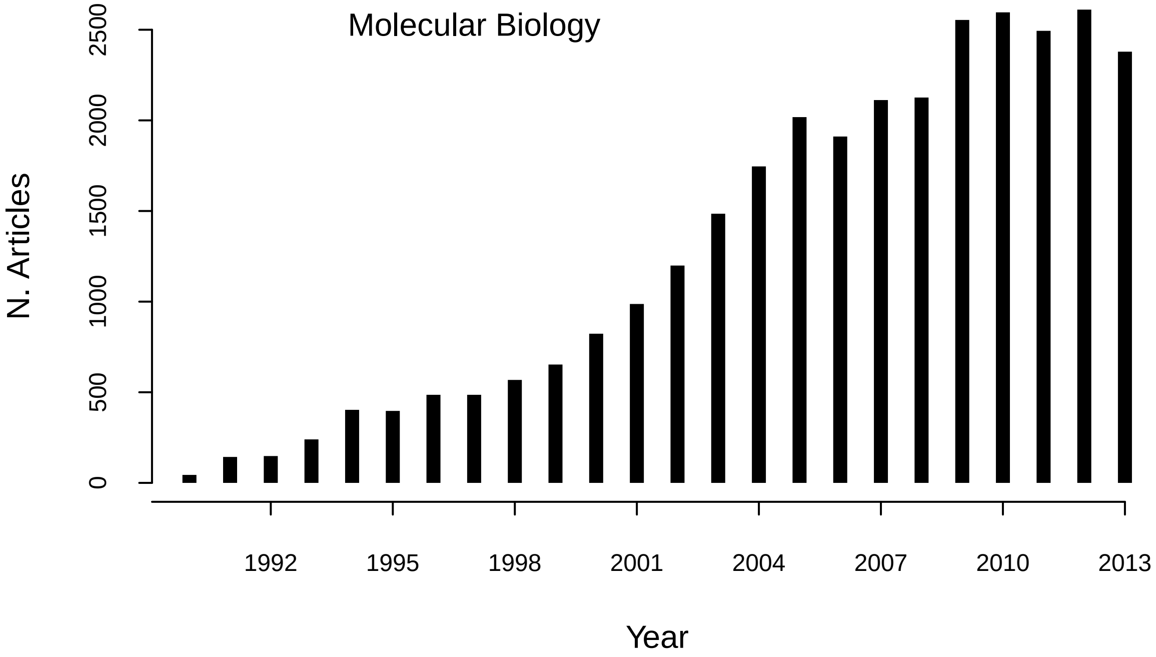

Figure 2: Molecular Biology data/random articles per year.

if upon running this script the quoted differences in medians (2 for NS, 1 for MB) might not add up, an exceptional sample might have been drawn and simply re-running this script should resolve the matter.)

Next, we establish if the observed increase in median article citation counts of the data over the random articles is statistically significant. We apply a (Wilcoxon) one-sided rank-sum test to determine the significance levels, both for Neuroscience and Molecular Biology.

```
wilcox.test(NS.data$TC, NS.rnd$TC, alternative="greater")
```

```
##
##  Wilcoxon rank sum test with continuity correction
##
## data:  NS.data$TC and NS.rnd$TC
## W = 11427000, p-value = 1.218e-14
## alternative hypothesis: true location shift is greater than 0
```

```
wilcox.test(MB.data$TC, MB.rnd$TC, alternative="greater")
```

```
##
##  Wilcoxon rank sum test with continuity correction
##
## data:  MB.data$TC and MB.rnd$TC
## W = 489150000, p-value < 2.2e-16
## alternative hypothesis: true location shift is greater than 0
```

To quantify the differences in medians in absolute terms, we measure the cumulative probability difference Δp at the median number of citations for random articles in both fields. This establishes how much more likely it is for data articles to receive the same number of citations as the field's overall average (Figures 3 and 4).

```
PlotLeftTail <- function (data.cites, rnd.cites, x.max=25) {
  # prepare the datasets
  data.ecdf = ecdf(data.cites)
  rnd.ecdf = ecdf(rnd.cites)
  med.cites = median(rnd.cites)
  # plot both CDFs
  plot(0:x.max, data.ecdf(0:x.max),
       type="l", col=2, frame.plot=F,
       xlab="Citations", ylab="CDF", ylim=c(0,1), cex.axis=.75)
  lines(0:x.max, rnd.ecdf(0:x.max), col=4)
  # describe p of data and rnd cites at the median of rnd cites
  p.data = data.ecdf(med.cites)
  p.rnd = rnd.ecdf(med.cites)
  lines(c(med.cites, med.cites),
        c(data.ecdf(med.cites), rnd.ecdf(med.cites)))
  text(med.cites + 5, y=rnd.ecdf(med.cites) - .2, cex=.75, label=bquote(
    P[rnd] (C == .(med.cites)) <= .(signif(p.rnd, digits=2))
  ))
  text(med.cites + 5, y=rnd.ecdf(med.cites) - .35, cex=.75, label=bquote(
    P[data] (C == .(med.cites)) <= .(signif(p.data, digits=2))
  ))
}
```

```
PlotLeftTail(NS.data$TC, NS.rnd$TC)
```

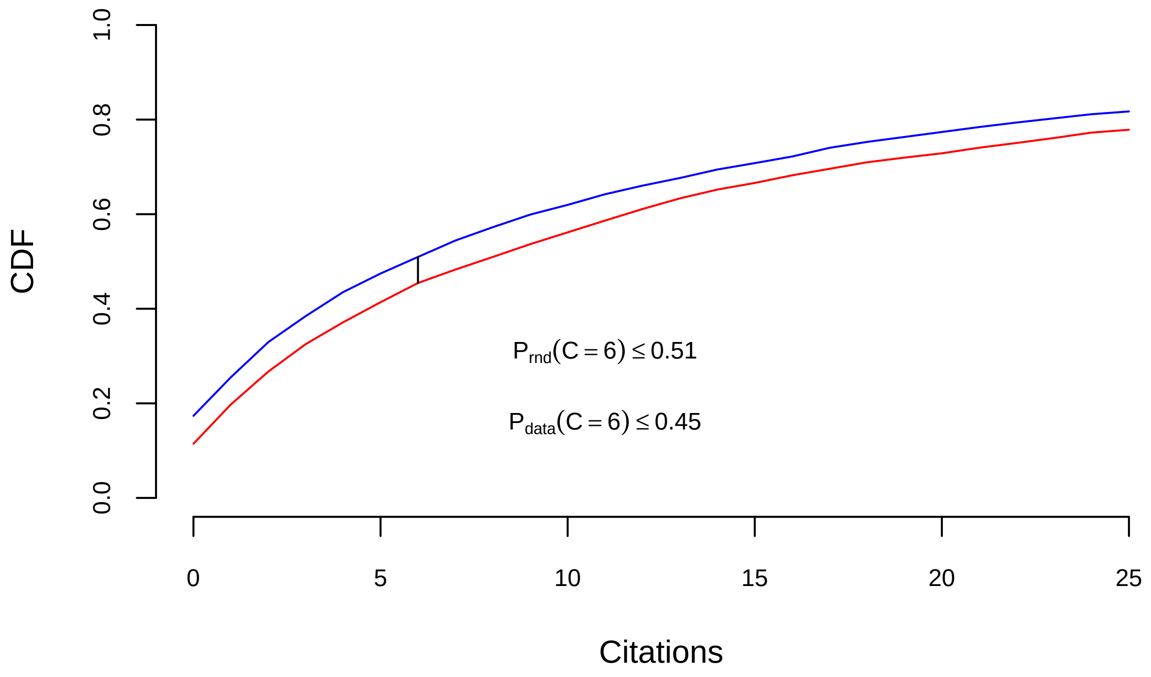

Figure 3: Neuroscience CDF, left tail. Red: for data article citations. Blue: for random article citations.

```
PlotLeftTail(MB.data$TC, MB.rnd$TC, 40)
```

Second, we compare the citation difference ΔC at $p = 0.1$ (i.e., in the last decile) in the complementary CDF. This quantifies the number of additional citations the top 10% of data articles typically receive when compared to the random population sample (Figures 5 and 6).

```
PlotHeavyTail <- function(data.cites, rnd.cites) {
  # prepare the datasets
  x.data = sort(unique(data.cites))
  x.rnd = sort(unique(rnd.cites))
  data.ecdf = ecdf(data.cites)
  rnd.ecdf = ecdf(rnd.cites)
  # plot both complementary CDFs (1 - CDF)
  # NB: log-log plot, so don't show the zero citation probability
  # NB: draws a cleaner, more legible Y-axis
  plot(x.data[x.data>0], 1.0 - data.ecdf(x.data[x.data<max(x.data)]),
       log="xy", ty="l", col=2, frame.plot=F,
       xlab="Citations", ylab="1 - CDF", yaxt="n", cex.axis=0.75)
  lines(x.rnd[x.rnd>0], 1.0 - rnd.ecdf(x.rnd[x.rnd<max(x.rnd)]), col=4)
  marks = c(1.0, 0.1, 0.01, 0.001, 1e-4, 1e-5, 1e-6, 1e-7, 1e-8, 1e-9)
  marks = marks[marks > 1-data.ecdf(max(x.data) - 1)]
  axis(2, at=marks, labels=marks, cex.axis=.75)
  # describe C at p == 0.9 and the number of rnd cites at that p
  delta.C = abs(quantile(data.ecdf, .9) - quantile(rnd.ecdf, .9))
  C.rnd = quantile(rnd.ecdf, .9)
  C.data = quantile(data.ecdf, .9)
```

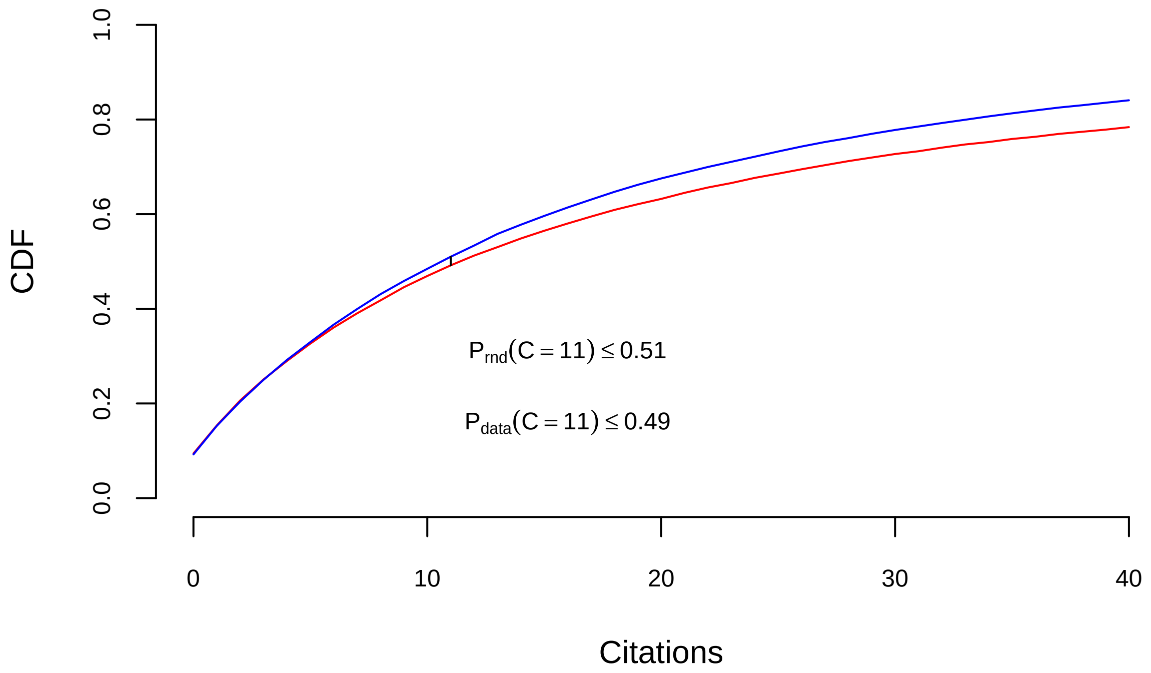

Figure 4: Molecular Biology CDF, left tail. Red: for data article citations. Blue: for random article citations.

```
  lines(c(quantile(data.ecdf, .9), quantile(rnd.ecdf, .9)), c(.1, .1))
  text(10, y=0.02, cex=.75, label=bquote(P[data] (C > .(C.data)) == 0.1))
  text(10, y=0.002, cex=.75, label=bquote(P[rnd] (C > .(C.rnd)) == 0.1))
}
```

```
PlotHeavyTail(NS.data$TC, NS.rnd$TC)
```

```
PlotHeavyTail(MB.data$TC, MB.rnd$TC)
```

Author-specific citation distributions

A data article citation index

One common way of ranking author citation impact today is the h-index, introduced by Hirsch (2005). However, as the data article sets are a limited selection of an author's works, the h-index would penalize highly cited authors who published data articles only once (their h-index would be 1) or a few times. In (2008), Bornmann showed that other index strategies can be more apt at predicting peer assessments than the h-index. Furthermore, Yong (2014) claims that the h-index does not constitute a significantly more accurate assessment than the total number of citations.

Therefore, summing up the number of citations for an author over all her publications seems more fair for the purpose of establishing an index for ranking the most prolific data-publishing authors. Nonetheless, two problems occur if we were to use the raw sum of citations.

1. A senior author who published her papers long ago is more likely to have accumulated more citations than a young scientist. However, the possible fact that a senior's data articles are still being cited should be factored in for a fair global ranking.

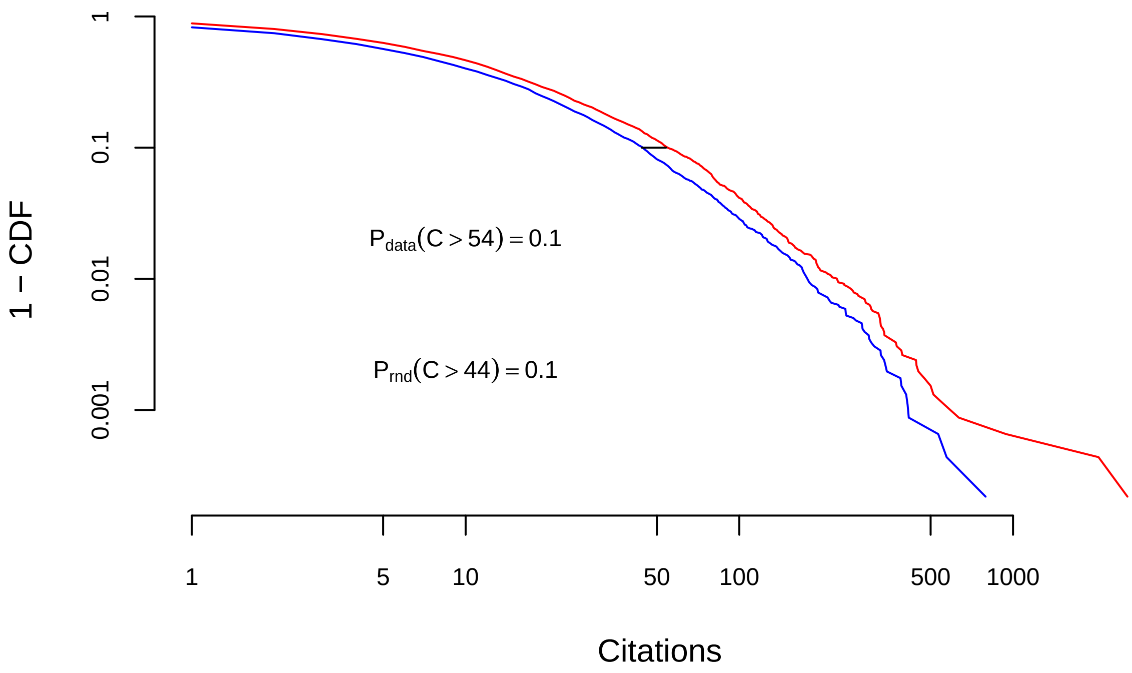

Figure 5: Complementary CDF showing the heavy tail of Neuroscience citations. Red: data article citations. Blue: random article citations.

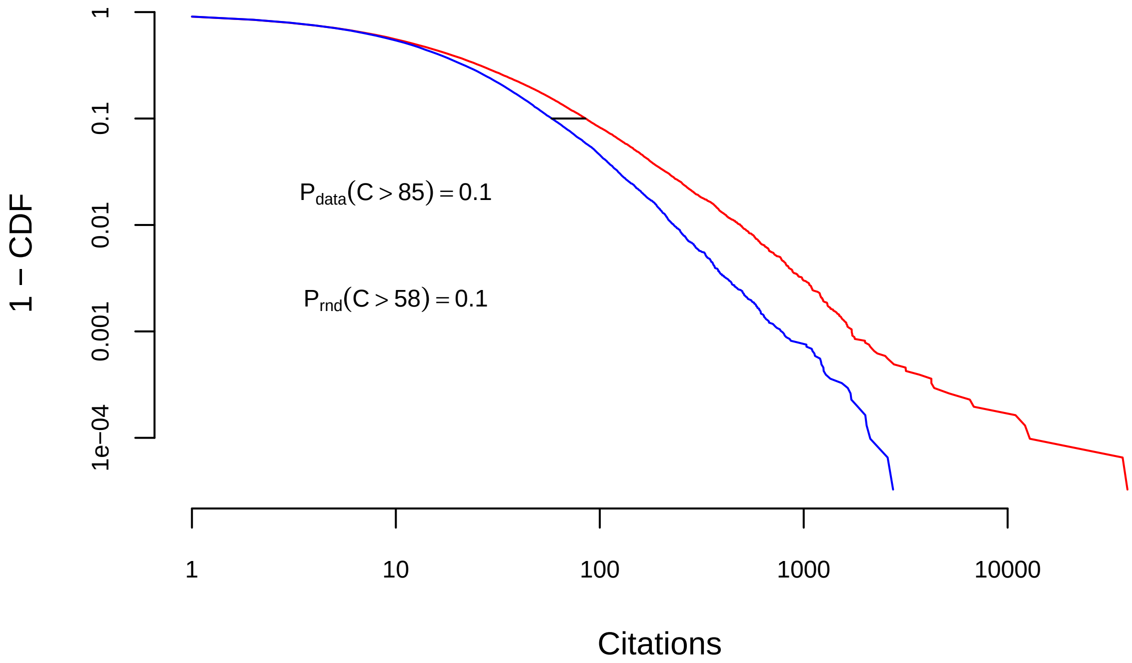

Figure 6: Complementary CDF showing the heavy tail of Molecular Biology citations. Red: data articles Blue: random articles.

2. Second, being the author of just one top-cited article could result in a larger sum (index rank) than being the author of many averagely cited articles due to the very heavy tails of the citation distributions. This implies that all co-authors of the most cited article(s) dominate the ranking.

Our intent therefore is to introduce a ranking that can mitigate these effects.

First, the datasets need to be transformed to calculate an author-centric citation index. The datasets contain *article citations*, i.e., the citation data are provided on a per-article basis. To extract the individual *author citations* from the datasets, we create an aggregate function to determine each author's personal set of articles.

```
AggregateCitations <- function(sample)
  aggregate(Citations~Author,
            data.frame(Author=unlist(sample$AU),
                       Citations=rep(sample$TC, sapply(sample$AU, length))),
            sum)

# helper function to split author name strings into individual authors
SplitAuthors <- function(authors) sapply(strsplit(authors, "; "), unique)
```

By summing up the citation counts of each neuroscientist's data articles, we get the following top-ten ranking:

```
NS.split <- data.frame(NS.data)
NS.split$AU <- SplitAuthors(NS.split$AU)
NS.agg <- AggregateCitations(NS.split)
head(NS.agg[with(NS.agg, order(-Citations)),], 10)
```

```
##                        Author Citations
## 19999     Williams, Brian A      2670
## 11854        McCue, Kenneth      2619
## 12642        Mortazavi, Ali      2619
## 16098     Schaeffer, Lorian      2619
## 20124          Wold, Barbara      2619
## 17121       Smith, Stephen M      2333
## 1161  Beckmann, Christian F      2255
## 1188   Behrens, Timothy E J      2124
## 8357         Jenkinson, Mark      2069
## 20226       Woolrich, Mark W      2062
```

Similarly, in Molecular Biology, we get:

```
##                Author Citations
## 1530  Altschul, S F     78500
## 49886   Lipman, D J     76777
## 56742     Miller, W     75933
## 59379    Myers, E W     45555
## 96903      Zhang, J     44075
## 28149       Gish, W     41236
## 52371   Madden, T L     38032
## 74701 Schaffer, A A     37551
## 97347      Zhang, Z     37218
## 89922       Wang, J     20384
```

The two top data articles in Molecular Biology are the publications of BLAST and PSI-BLAST by Altschul et al., each having approximately 40 thousand citations. If those two papers are removed from the set (as in the next ranking below), the ranking changes substantially. As can be seen below, the nine co-authors[7] of those two most cited data papers occupied all the top ranks in the ranking shown above:

```
# exclude the BLAST and PSI-BLAST papers:
MB.agg <- AggregateCitations(MB.split[3:nrow(MB.split),])
head(MB.agg[with(MB.agg, order(-Citations)),], 10)
```

```
##              Author Citations
## 89922     Wang, J     20384
## 43759 Koonin, E V     19161
## 6855    Birney, E     16968
## 36082  Hubbard, T     16905
## 21306   Durbin, R     16372
## 369    Adams, M D     16098
## 2490   Aravind, L     14857
## 6041  Berman, H M     14499
## 7976      Bork, P     14405
## 32603 Haussler, D     14282
```

It should be noted that there is no perceivable reason to exclude these two papers from the list of database papers. Rather, this Gedankenspiel should show how brittle a pure "sum of citations" approach would be with regard to minor fluctuations in the sample.

One problem that can be observed - and about which little can be done here - is the author first and middle name abbreviation problem. For example, most of "P. Bork's" and "Peer Bork's" or "A. Bairoch's" and "Amos Bairoch's" counts probably should be aggregated. But it is hard to judge if there is no other scientist with the same initials, and it is impossible to fully separate those assignments based on the data we can get from Thomson Reuter's alone.

To partially address this matter, we abbreviate all first and middle names using two regular expressions, thereby effectively normalizing all names. This choice implies that authors with many publications are better represented in our results, because their index value is no longer divided between their abbreviated and full name. It comes at the cost of sacrificing the ability to reliably find the correct index value of mid- and low-ranking authors with their full name if their (first and middle name) initials happen to coincide with a higher-ranked author. While top authors therefore will have artificially increased counts from coinciding names, this increase can be expected to be proportionally smaller due to the power-law behavior of citation counts. A remaining problem is top authors whose names coincide or if they have very common names. However, as this index only considers data authors, the absolute number of possible name collisions is proportionally lower than for the set of names from the entire scientific bibliome.

```
NormalizeFirstNames <- function(name_lists)
  lapply(name_lists, function(authors)
    gsub(", ([A-Z])[a-z]+[ -]", ", \\1 ",
         gsub("[ -]([A-Z])[a-z]+$", " \\1",
              authors)))
```

To dampen the dominant effect of the most cited papers, it is more appropriate to calculate a sum of logs instead of the sum (Radev et al. 2009) to establish a **data article citation index** (***DAC*-index**). This can be further justified by the fact that the log-normal is a valid distribution model for citation counts, as discussed earlier.

[7] That is: Altschul, S F; Gish, W; Miller, W; Myers, E W; Lipman, D J, Altschul, S F; Madden, T L; Schaffer, A A; Zhang, J; Zhang, Z; Miller, W; Lipman, D J

$$D = log \prod n_i = \sum log\ n_i$$

Where n_i is the citation count for data article i by the author being indexed. Note that any base will do for the logarithm, as it does not influence the relative ordering of authors.

One effect of this log-based index is that it gives authors of many medium-impact papers an edge over authors only appearing on the one or two most cited papers. Another good reason for using a sum of logs is that it is more favorable towards authors that might be working in a sub-discipline that gains relatively less citations (e.g., if she is working on some rare model organism). A third effect is that an author with many papers with only very few citations each can accumulate citation impact, too. While that last issue might not immediately appear as desired, the actual work required to create that many publications should be acknowledged to some extent; Not the least because paradigm-changing work can sometimes go unnoticed for years and even decades without picking up a justified number of citations (Wang, Song, and Barabási 2013). Finally, in comparison to more elaborate procedures, using the sum of logs is a simple calculation and therefore follows the principle of Occam's razor.

Returning to the two problems stated in the beginning of this section, the DAC-index solves both: A senior scientist still can more easily accumulate more citation impact, but due to log-scaling and the fact that newer articles generally receive more citations, it is easier for young scientists to catch up. And only being a co-author of the top cited article(s) without any further data publications is no longer sufficient to dominate the ranking.

This leads to the following, final **DAC-index ranking for Neuroscience**:

```
NS.agg <- data.frame(AU=I(NS.split$AU), TC=log(NS.split$TC, 2), PY=NS.split$PY)
NS.agg$AU <- NormalizeFirstNames(NS.agg$AU)
NS.agg <- AggregateCitations(NS.agg)
NS.ranking <- NS.agg[with(NS.agg, order(-Citations)),]
head(NS.ranking, 20)
```

```
##                  Author Citations
## 17165        Toga, A W 113.12707
## 10692      Marmarou, A 108.01908
## 11904      Murray, G D  76.32372
## 10344      Maas, A I R  68.02451
## 16362  Steyerberg, E W  67.02451
## 10219            Lu, J  66.45297
## 3952       DeVivo, M J  62.37555
## 2167        Butcher, I  60.28305
## 11037      McHugh, G S  60.28305
## 17727   Van Essen, D C  57.99922
## 10717    Marshall, L F  55.54335
## 17066    Thompson, P M  53.89455
## 5194      Foulkes, M A  50.69958
## 6006         Gordon, E  47.96464
## 11961        Nagase, T  47.43339
## 18642    Williams, R W  47.33676
## 4037       Diener, H C  46.86433
## 11543        Mohr, J P  46.40805
## 8976         Kotter, R  44.47459
## 11916 Mushkudiani, N A  43.89941
```

Applying the same methodology to **Molecular Biology** authors give the following **DAC-index ranking**:

```
##                  Author Citations
## 3426         Bairoch, A  685.2188
## 37179        Koonin, E V  365.7870
## 18379        Dunker, A K  307.6099
## 18483          Durbin, R  305.4136
## 61863            Sali, A  271.6663
## 66334        Skolnick, J  253.7186
## 29690 Hochstrasser, D F  235.2157
## 62123       Sanchez, J C  229.1041
## 2154          Appel, R D  226.7728
## 73119       Uversky, V N  222.6697
## 3461            Baker, D  213.0058
## 12233         Chothia, C  208.2536
## 19007       Eisenberg, D  198.0262
## 8661           Bucher, P  178.5349
## 40901          Levitt, M  174.9632
## 61374      Rychlewski, L  173.0159
## 52054       Obradovic, Z  170.4503
## 26038       Gromiha, M M  168.5819
## 33474        Jones, D T  165.3952
## 64955          Shen, H B  163.1558
```

The chosen cutoff of showing the top 20 ranks was made arbitrary.

Data author comparisons

Building on the DAC-index result, we establish if the data articles have an significantly stronger citation impact compared to the author's other articles. (Note that to ensure a fair comparison the selected articles are exclusively from the author's respective field (NS or MB), and do not include her articles from any other field.)

```
CompareAuthor <- function (all.articles, data, author.name) {
  # Extract the data articles from an author's article set
  data.articles = semi_join(all.articles, data, by="PM")
  other.articles = anti_join(all.articles, data, by="PM")
  # Apply a one-sided rank-sum test and report
  test.result = wilcox.test(data.articles$TC, other.articles$TC,
                            alternative="greater", exact=F)
  cat(author.name, "\n")
  cat("median citation counts other =", median(other.articles$TC),
      "from", length(other.articles$TC), "articles\n")
  cat("median citation counts data  =", median(data.articles$TC),
      "from", length(data.articles$TC), "articles\n")
  cat("one-sided rank-sum p-value   =", test.result$p.value, "\n")

  # Visualize the two distributions as side-by-side box-plots
  # NB: has to be calculated before modifying the Os!
  mean.other = mean(other.articles$TC)
  mean.data = mean(data.articles$TC)
  # Modify Os to allow for a log-scaled boxplot with zero-citations
  other.articles$TC[other.articles$TC==0] = 0.1
  data.articles$TC[data.articles$TC==0] = 0.1
  boxplot(other.articles$TC, data.articles$TC,
```

```
         names=c("Other", "Data"), notch=F, log="y", las=2,
         ylab="Citations",
         yaxt="n", cex.axis=0.7)
  # Add x-mark of the mean of the distributions
  points(1, mean.other, pch=4)
  points(2, mean.data, pch=4)
  # Add author name as title, with test significance stars
  stars = symnum(test.result$p.value, corr=F,
                 cutpoints = c(0,  .001, .01, .05, .1, 1),
                 symbols = c("***","**","*","."," "))
  title(paste0(author.name, stars[[1]]))

  # Calculate and draw a clean citation count axis
  ymin = floor(log(min(data.articles$TC, other.articles$TC), 10))
  ymax = ceiling(log(max(all.articles$TC), 10))
  yseq = seq(ymin, ymax, length.out=6)
  ylabs = format(10^yseq, trim=T, scientific=F, digits=0, format="f")
  axis(2, at=10^yseq, labels=ylabs)
}
```

In Figures 7 and 8, the side-by-side box plots of each of the top ten authors in both fields are shown. Based on a one-sided Wilcoxon rank-sum test, we establish if the differences between their entire citation sets and “other” (non-data) citation count distributions are statistically significant. This is indicated by the asterisk/star notation of significance levels (*p $<$ 0.05, **p $<$ 0.01, ***p $<$ 0.001) in the author names (titles). In other words, the data citation impact of authors that are “decorated with stars” is significantly stronger than their other, non-data article citation impact. The difference is quantified by the two medians below.

Neuroscience authors:

```
par(mfrow=c(2,5))
for (name in names(NS.aut)) {
  CompareAuthor(NS.aut[[name]], NS.data, name)
}
```

```
## Butcher
## median citation counts other = 18.5 from 16 articles
## median citation counts data  = 79 from 10 articles
## one-sided rank-sum p-value   = 0.002033657

## DeVivo
## median citation counts other = 24 from 84 articles
## median citation counts data  = 56 from 15 articles
## one-sided rank-sum p-value   = 0.02042069

## Lu
## median citation counts other = 14 from 171 articles
## median citation counts data  = 79 from 10 articles
## one-sided rank-sum p-value   = 0.0001250827

## Maas
## median citation counts other = 18.5 from 74 articles
## median citation counts data  = 79 from 12 articles
## one-sided rank-sum p-value   = 0.0008404096
```

```
## McHugh
## median citation counts other = 25 from 11 articles
## median citation counts data  = 79 from 10 articles
## one-sided rank-sum p-value   = 0.01885458

## Marmarou
## median citation counts other = 26 from 199 articles
## median citation counts data  = 80 from 17 articles
## one-sided rank-sum p-value   = 0.0001465897

## Murray
## median citation counts other = 33 from 86 articles
## median citation counts data  = 81 from 13 articles
## one-sided rank-sum p-value   = 0.01556908

## Steyerb.
## median citation counts other = 19.5 from 90 articles
## median citation counts data  = 81 from 11 articles
## one-sided rank-sum p-value   = 4.62999e-05

## Toga
## median citation counts other = 29 from 522 articles
## median citation counts data  = 21 from 31 articles
## one-sided rank-sum p-value   = 0.9149317

## Van Essen
## median citation counts other = 65 from 130 articles
## median citation counts data  = 67 from 10 articles
## one-sided rank-sum p-value   = 0.5817354
```

```
par(mfrow=c(1,1))
```

Molecular Biology authors:

```
par(mfrow=c(2,5))
for (name in names(MB.aut)) {
  CompareAuthor(MB.aut[[name]], MB.data, name)
}
```

```
## Appel
## median citation counts other = 32 from 34 articles
## median citation counts data  = 33.5 from 44 articles
## one-sided rank-sum p-value   = 0.2386653

## Bairoch
## median citation counts other = 52 from 47 articles
## median citation counts data  = 88.5 from 110 articles
## one-sided rank-sum p-value   = 0.01216537

## Dunker
## median citation counts other = 30.5 from 124 articles
## median citation counts data  = 54.5 from 54 articles
## one-sided rank-sum p-value   = 0.001519785
```

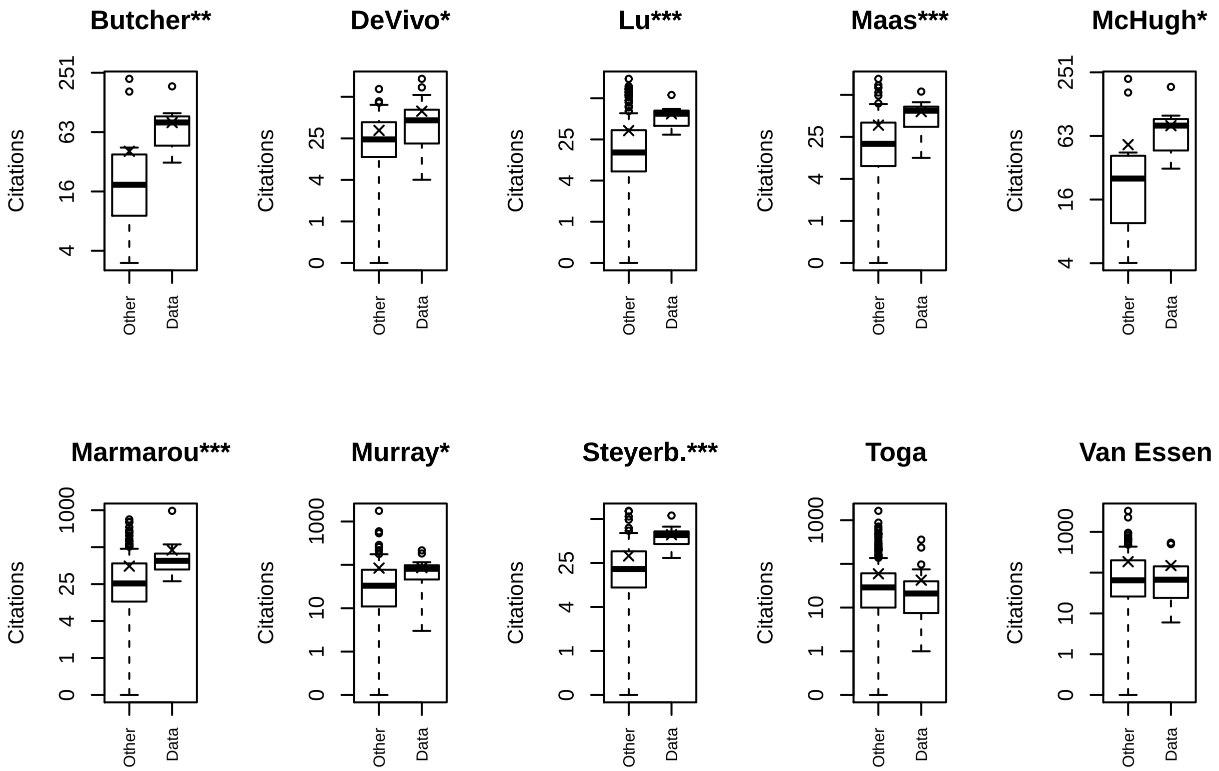

Figure 7: Box-and-whisker plots of data vs. other (non-data) citation count distributions for the top-ten Neuroscience data authors. Title asterisks: rank-sum test significance levels (`*p<0.05`; `**p<0.01`; `***p<0.001`). Additional x-mark: sample mean. Whisker sizes: 1.5 interquartile ranges.

```
## Durbin
## median citation counts other = 78.5 from 64 articles
## median citation counts data  = 148 from 44 articles
## one-sided rank-sum p-value   = 0.008748146

## Hochstr.
## median citation counts other = 22.5 from 146 articles
## median citation counts data  = 55 from 46 articles
## one-sided rank-sum p-value   = 0.001811152

## Koonin
## median citation counts other = 55 from 444 articles
## median citation counts data  = 89.5 from 56 articles
## one-sided rank-sum p-value   = 0.0009783556

## Sali
## median citation counts other = 35 from 227 articles
## median citation counts data  = 42 from 52 articles
## one-sided rank-sum p-value   = 0.1638974

## Sanchez
## median citation counts other = 24 from 133 articles
## median citation counts data  = 59 from 41 articles
## one-sided rank-sum p-value   = 0.001213024

## Skolnick
## median citation counts other = 28 from 154 articles
## median citation counts data  = 42 from 49 articles
## one-sided rank-sum p-value   = 0.01283627

## Uversky
## median citation counts other = 26.5 from 258 articles
## median citation counts data  = 47 from 43 articles
## one-sided rank-sum p-value   = 0.02428859
```

```r
par(mfrow=c(1,1))
```

Note that it might seem appealing to ask if data articles play a significant role in an author's overall citation impact. However, that is a poorly formulated hypothesis, as it depends more on the relative fraction of data articles than on the specific citation counts. To put it in plain words, adding a few outliers with otherwise mostly similar data should not have a significant impact on the median. In fact, only Bairoch's overall citation impact significantly increases because of his data articles, while all other authors' does not (data not shown). Which can be easily predicted from the fact that only Bairoch has more data articles than other, non-data articles (and with McHugh being the obvious, second-best candidate).

An online DAC-index

As the citation counts from Thomson Reuters ISI WoK cannot be accessed programmatically, the data had to be downloaded manually in batches of 500 citations. Therefore, it only was possible to establish a static DAC-index for the purpose of this publication. In the future, if Thomson Reuters were to provide open (web-) access to their citation data for public, scientific purposes, it would be possible to generate an online, continuosly up-to-date version of this index.

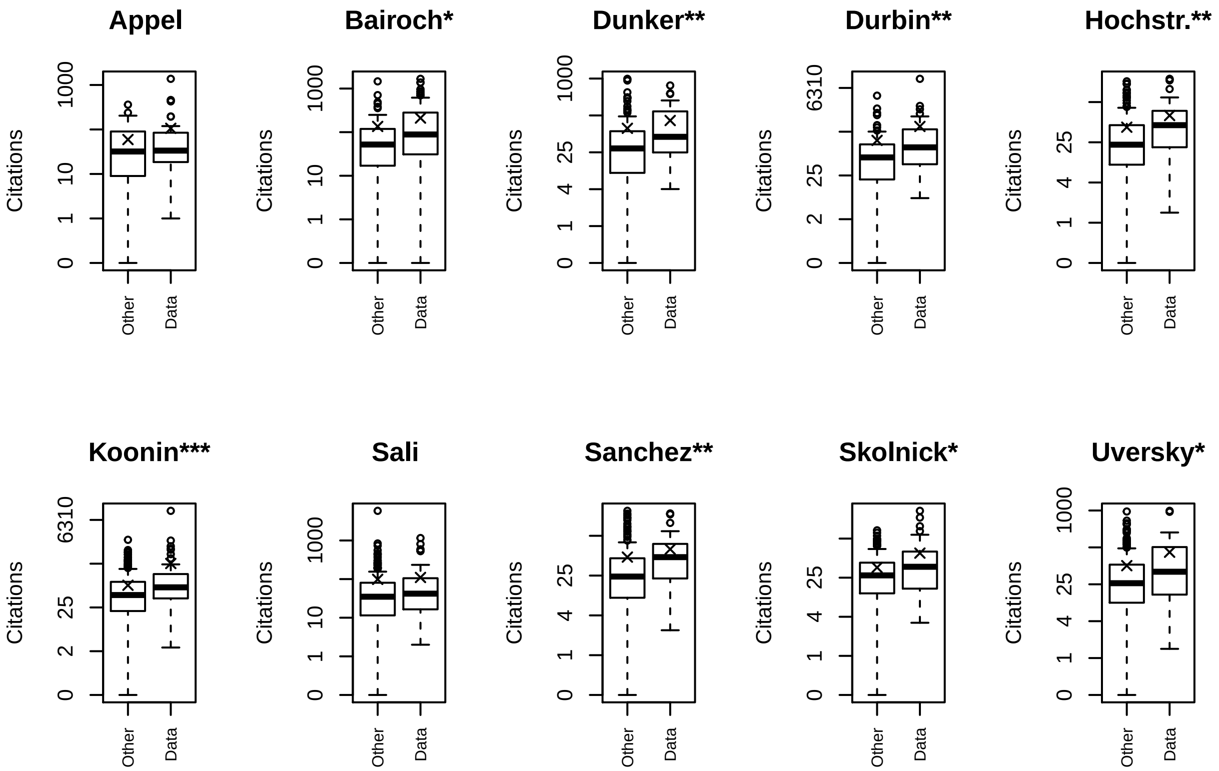

Figure 8: Box-and-whisker plots of data vs. other (non-data) citation count distributions for the top-ten Molecular Biology data authors. Title asterisks: rank-sum test significance levels (*p<0.05; **p<0.01; ***p<0.001). Additional x-mark: sample mean. Whisker sizes: 1.5 interquartile ranges.

References

Bornmann, Lutz, Rüdiger Mutz, and Hans-Dieter Daniel. 2008. “Are There Better Indices for Evaluation Purposes Than the H Index? A Comparison of Nine Different Variants of the H Index Using Data from Biomedicine.” *Journal of the American Society for Information Science and Technology* 59 (5): 830–37.

Hirsch, Jorge E. 2005. “An Index to Quantify an Individual’s Scientific Research Output.” *Proceedings of the National Academy of Sciences of the United States of America* 102 (46): 16569–72.

Radev, Dragomir R., Mark Thomas Joseph, Bryan Gibson, and Pradeep Muthukrishnan. 2009. “A Bibliometric and Network Analysis of the Field of Computational Linguistics.” *Journal of the American Society for Information Science and Technology* 1001: 48109–41092.

Redner, S. 1998. “How Popular Is Your Paper? An Empirical Study of the Citation Distribution.” *The European Physical Journal B - Condensed Matter and Complex Systems* 4 (2): 131–34.

Shockley, William. 1957. “On the Statistics of Individual Variations of Productivity in Research Laboratories.” *Proceedings of the IRE* 45 (3): 279–90.

Solla Price, Derek J. de. 1965. “Networks of Scientific Papers.” *Science* 149 (3683): 510–15.

Stringer, Michael J., Marta Sales-Pardo, and Luís A. Nunes Amaral. 2010. “Statistical Validation of a Global Model for the Distribution of the Ultimate Number of Citations Accrued by Papers Published in a Scientific Journal.” *Journal of the American Society for Information Science and Technology* 61 (7): 1377–85.

Stringer, Michael J., Marta Sales-Pardo, and Luís A. Nunes Amaral. 2008. “Effectiveness of Journal Ranking Schemes as a Tool for Locating Information.” *PLoS ONE* 3 (2): e1683.

Wallace, Matthew L., Vincent Larivière, and Yves Gingras. 2009. “Modeling a Century of Citation Distributions.” *Journal of Informetrics* 3 (4): 296–303.

Wang, Dashun, Chaoming Song, and Albert-László Barabási. 2013. “Quantifying Long-Term Scientific Impact.” *Science* 342 (6154): 127–32.

Yong, Alexander. 2014. “A Critique of Hirsch’s Citation Index: A Combinatorial Fermi Problem.” *Notices of the American Mathematical Society* 61 (9): 1040.
